# Design, Synthesis and Biological Evaluation of 6-(2,6-Dichloro-3,5-dimethoxyphenyl)-4-substituted-1*H*-indazoles as Potent Fibroblast Growth Factor Receptor Inhibitors

**DOI:** 10.3390/molecules21101407

**Published:** 2016-10-22

**Authors:** Zhen Zhang, Dongmei Zhao, Yang Dai, Maosheng Cheng, Meiyu Geng, Jingkang Shen, Yuchi Ma, Jing Ai, Bing Xiong

**Affiliations:** 1 Key Laboratory of Structure-Based Drug Design & Discovery of Ministry of Education, Shenyang Pharmaceutical University, Shenyang 110016, China; 18204012193@163.com (Z.Z.); mscheng@263.net (M.C.); 2 Department of Medicinal Chemistry, State Key Laboratory of Drug Research, Shanghai Institute of Materia Medica, Chinese Academy of Sciences, 555 Zuchongzhi Road, Shanghai 201203, China; jkshen@simm.ac.cn; 3 Division of Anti-Tumor Pharmacology, State Key Laboratory of Drug Research, Shanghai Institute of Materia Medica, Chinese Academy of Sciences, 555 Zuchongzhi Road, Shanghai 201203, China; daiyang@simm.ac.cn (Y.D.); mygeng@simm.ac.cn (M.G.)

**Keywords:** cancer, FGFR, inhibitor, 4-substituted-1*H*-indazole

## Abstract

Tyrosine kinase fibroblast growth factor receptor (FGFR), which is aberrant in various cancer types, is a promising target for cancer therapy. Here we reported the design, synthesis, and biological evaluation of a new series of 6-(2,6-dichloro-3,5-dimethoxyphenyl)-4-substituted-1*H*-indazole derivatives as potent FGFR inhibitors. The compound 6-(2,6-dichloro-3,5-dimethoxyphenyl)-*N*-phenyl-1*H*-indazole-4-carboxamide (**10a**) was identified as a potent FGFR1 inhibitor, with good enzymatic inhibition. Further structure-based optimization revealed that 6-(2,6-dichloro-3,5-dimethoxyphenyl)-*N*-(3-(4-methylpiperazin-1-yl)phenyl)-1*H*-indazole-4-carboxamide (**13a**) is the most potent FGFR1 inhibitor in this series, with an enzyme inhibitory activity IC_50_ value of about 30.2 nM.

## 1. Introduction

Fibroblast growth factor receptors (FGFRs), consisting of the four members FGFR1-4, belong to the family of receptor tyrosine kinases (RTKs) and play fundamental roles in several basic biological processes, including tissue development, angiogenesis and tissue regeneration [1,2,3]. Overactivation of FGFR signaling occurs in many types of cancer due to gene amplification, mutations or translocations [4,5,6,7]. Aberrant FGFR signaling drives oncogenic growth of tumor subsets, especially those lacking effective treatments, such as 20% squamous non-small cell lung carcinoma and 4% triple-negative breast cancer, etc. [8,9,10,11]. Therefore, FGFR has been validated as an attractive target for targeted cancer therapy [8,12,13,14].

**Figure 1:**
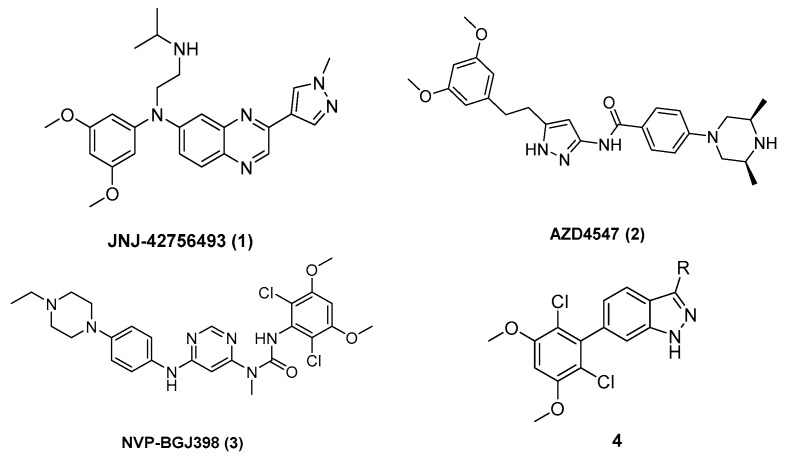
Structures of some representative selective FGFR inhibitors.

In recent years, several small molecule FGFR inhibitors have been reported, and some of them are now in clinical trials [15]. The early examples of FGFR inhibitors are predominantly multi-targeting drugs, such as nintedanib, lenvatinib, dovitinib, and lucitanib [16]. Although simultaneous inhibition of multiple RTKs may reinforce the efficacy in patients by concomitant disturbance of redundant pathways, it may also increase the chance of side effects or severe toxicity [17,18]. Thus, discovery of potent and selective FGFR inhibitors is an urgent need in cancer treatment. Currently, several FGFR-selective inhibitors have progressed into clinical trials (Figure 1) [1,2,3,4,5,6,7,8,9,10,11,12,13,14,15,16,17,18,19,20,21]. JNJ-42756493 (**1**) is a potent oral pan-FGFR inhibitor with IC_50_ values in the low nanomolar range for FGFR1-4 and it was effective in vitro and in vivo in CRC tumors such as cell line NCI-H716 harboring amplified FGFR2 [19]. AZD4547 (**2**) is a highly potent and selective FGFR1-3 inhibitor. During the phase I trial, on-target activity was observed in five of 20 patients with tumors harboring FGFR signaling aberrations. Better efficacy was shown in patients with a high level of FGFR amplification [20]. NVP-BGJ398 (**3**), currently in phase II clinical trials, inhibits FGFR1-4 with low nanomolar potency at the molecular level (IC_50_ = 0.9, 1.4, 1.0, and 60 nM, respectively) and demonstrated at least 200-fold selectivity for FGFRs over other evaluated kinases [21].

Recently, several series of 6-(2,6-dichloro-3,5-dimethoxyphenyl)-1*H*-indazole compounds **4** were reported as FGFR inhibitors [22,23]. Based on this promising scaffold and excellent bioactivity, we continued to investigate this chemotype in detail. Since the reported 6-(2,6-dichloro-3,5-dimethoxyphenyl)-1*H*-indazole compounds all focused on the optimization at C3-position of indazole that extends into the solvent part in the FGFR binding site, we were wondering whether another position could be utilized to increase the binding activity. By taking advantage of docking studies, we hypothesized that the C4-position of indazole may be a new direction for optimization, as it can extend into a new binding subpocket in the ATP site of FGFR. Therefore, we designed and synthesized a new series of FGFR inhibitors containing 6-(2,6-dichloro-3,5-dimethoxyphenyl)-1*H*-indazole scaffold, and the FGFR1 enzymatic evaluation indeed showed the good bioactivity of this series of inhibitors.

## 2. Results and Discussion

### 2.1. Chemistry

**Scheme 1:**
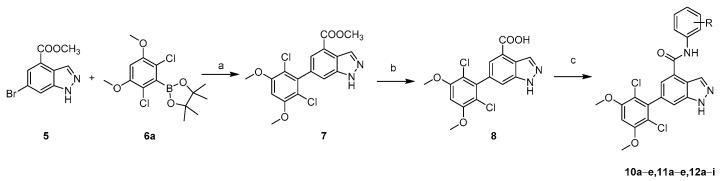
Synthesis of 1*H*-indazole-4-carboxamide derivatives **10a**–**e**, **11a**–**e**, **12a**–**i**. Reagents and conditions: (**a**) (i) Boc_2_O, Cs_2_CO_3_, 1,4-dioxane, r.t., 0.5 h; (ii) 2-(2,6-dichloro-3,5-dimethoxyphenyl)-4,4,5,5-tetramethyl-1,3,2-dioxaborolane, Pd(dppf)Cl_2_, 1,4-dioxane:H_2_O = 3:1, 100 °C, 2 h, 62.3%; (**b**) LiOH·H_2_O, THF, 50 °C, 6 h, 76.2%; (**c**) (i) POCl_3_, pyridine, 0 °C, 1 h; (ii) aniline, 0 °C, 10 min; (iii) r.t., 1.5 h, 60.5%–82.3%.

Compounds **10a**–**e**, **11a**–**e**, **12a**–**i** were prepared according to the procedure shown in Scheme 1. Suzuki coupling of methyl 6-bromo-1*H*-indazole-4-carboxylate (**5**) with 2-(2,6-dichloro-3,5-dimethoxyphenyl)-4,4,5,5-tetramethyl-1,3,2-dioxaborolane (**6a**) provided methyl 6-(2,6-dichloro-3,5-dimethoxyphenyl)-1*H*-indazole-4-carboxylate (**7**). Treatment of compound **7** with lithium hydroxide afforded 6-(2,6-dichloro-3,5-dimethoxyphenyl)-1*H*-indazole-4-carboxylic acid (**8**). Compounds **10a**–**e**, **11a**–**e**, **12a**–**i** were prepared by subjecting compound **8** to condensation with the appropriate aromatic amine derivatives.

**Scheme 2:**
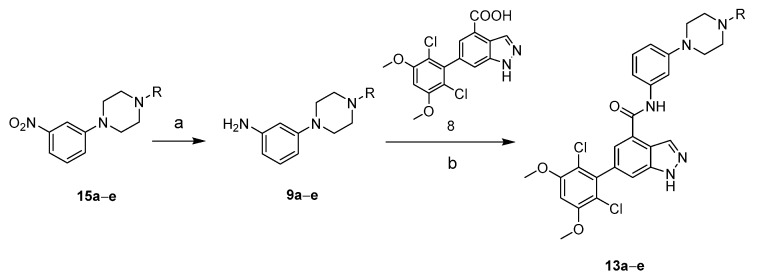
Synthesis of 1*H*-indazole-4-carboxamide derivatives **13a**–**e**. Reagents and conditions: (**a**) Fe, NH_4_Cl, ethanol, 80 °C, 2–6 h, 35.5%–55.1%; (**b**) HATU, DIPEA, DCM, r.t., 3 h, 55.6%–65.8%.

Compounds **13a**–**e** were prepared according to the procedure shown in Scheme 2. Commercially available **15a**–**e** were subjected to a reduction reaction with iron powder to afford 3-(4-substituted-piperazin-1-yl)anilines **9a**–**e**. Compounds **13a**–**e** were prepared by condensation of compound **8** with compounds **9a**–**e**.

### 2.2. Inhibitor Design

**Figure 2:**
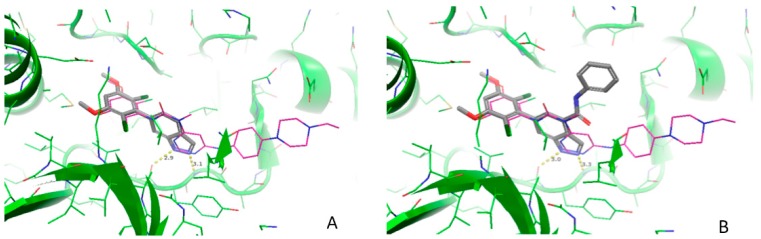
The predicted binding conformation of compound **4** (**A**) and **10a** (**B**) in the ATP binding site of FGFR1 based on the docking studies.

To elucidate the interaction of the 6-(2,6-dichloro-3,5-dimethoxyphenyl)-1*H*-indazole scaffold **4** with FGFR, we performed a docking study on compound **4** in the ATP site of FGFR1 using a reported crystal structure of the FGFR1 kinase domain (PDB ID: 3TT0), as shown in Figure 2A. According to this model, the NH at the N1-position of 1*H*-indazole is involved in a critical H-bond with the GLU562 carbonyl, and the N2 atom of the indazole ring forms another hydrogen bond with residue ALA564 located at the hinge region of the ATP-binding pocket. In addition, the two chlorine atoms, which formed favorable hydrophobic contacts with Val561 and Ala640, respectively, force the tetrasubstituted phenyl ring to adopt an almost perpendicular orientation with respect to the plane of the indazole ring. Overall the tetrasubstituted phenyl ring superimposes well with the NVP-BGJ398 in the 3TT0 co-crystal structure. Through this detailed analysis of the predicted bond conformation of compound **4**, it was clear that the indazole C3 is an obvious direction for optimization as indicated by NVP-BGJ398. We also noticed that the methyl group at the amide moiety of NVP-BGJ398 is pointing to a subpocket, which could also be utilized for optimization. Therefore, we designed compound **10a** and docked it into the FGFR1 binding site. As shown in Figure 2B, the 6-(2,6-dichloro-3,5-dimethoxyphenyl)-1*H*-indazole scaffold still remains at the same position by forming the essential interactions described before, while the substituted benzyl amide group extends upward into a subpocket. Considering this interesting binding mode, we synthesized this compound and evaluated its activity in the FGFR1 enzymatic assay. The result confirmed that this compound **10a** has good activity. Next, we selected compound **10a** as the starting point for further modification.

### 2.3. The Structure–Activity Relationship of Indazole Derivatives

We first examined the impact of substitutions at the *meta*- or *para*-positions of the phenyl ring of compound **10a** on the biological activity against FGFR1 (Table 1). Compared with compound **10a**, introduction of an acetyl group at the *meta*-position of phenyl ring (compound **10b**) caused a slight increase in inhibitory activity, while introducing an acetyl at the *para*-position (compound **10c**) decreased the inhibitory activity. This trend is also reflected from the pair of compounds with methoxyl at the *meta*-position (**10d**) and *para*-position (**10e**). This might imply that substitution of the *meta*-position of phenyl ring could form favorable interactions with the binding site.

The designed and synthesized compounds **11a**–**e** with modifications on the phenyl moiety of compound **10d** were assessed for their FGFR1 inhibitory activity (Table 2). The results indicated that replacement of the phenyl ring (compound **10d**) with pyridines (compounds **11a**–**c**) or pyrimidines (compounds **11d**,**e**) slightly decreased the activity. According to the obtained structure-activity relationship, we then prepared various compounds **12a**–**i** with substitutions at the C3-position of the phenyl ring and evaluated them in the enzymatic assay (Table 3). Incorporation of methoxycarbonyl (**12a**), ethoxycarbonyl (**12b**), nitro (**12d**), and 3-methyl-2-oxoimidazolidin-1-yl (**12e**) groups all reduced the enzymatic potency. Fortunately, compound containing a methylcarbamoyl at the phenyl 3-position (**12b**, IC_50_ = 38.6 ± 0.2 nM) showed better inhibitory activity than **10a** (IC_50_ = 69.1 ± 19.8 nM).

Meanwhile, it was also found that incorporation of morpholine (**12f**) and piperazine (**12g**) could retain the enzymatic potency. However, compounds containing aromatic structures at the *meta*-position of phenyl displayed substantially reduced inhibitory activities.

In order to further improve the enzymatic potency of this series of compounds against FGFR1, various groups were incorporated at the 4’-position of the piperazine of compound **12g** (Table 4). Compared to **12g**, most of the resulting analogues **13b**–**e** showed diminished enzymatic activities. Nevertheless, the 4-methylpiperazine analogue **13a** demonstrated excellent inhibitory activity in the enzymatic assay (IC_50_ = 30.2 ± 1.9 nM).

## 3. Experimental Section

### 3.1. General Information

^1^H-NMR (400 MHz) and ^13^C-NMR (125 MHz) spectra were recorded by using a Mercury-400 and a Mercury-500 High Performance Digital FT-NMR spectrometer, respectively (Varian, Palo Alto, CA, USA) with tetramethylsilane (TMS) as an internal standard. Abbreviations for peak patterns in NMR spectra are as follows: s = singlet, d = doublet, and m = multiplet. Low-resolution mass spectra were obtained with a LCQ Deca XP mass spectrometer (Finnigan, Palo Alto, CA, USA) using a CAPCELL PAK C18 (50 mm × 2.0 mm, 5 ZM) or an Agilent ZORBAX Eclipse XDB C18 (50 mm × 2.1 m, 5 ZM) in positive or negative electrospray mode. Low-resolution mass spectra and high-resolution mass spectra were recorded at an ionizing voltage of 70 eV on a Finnigan/MAT95 spectrometer. Compound purity was determined by analytical HPLC (Gilson, Middleton, WI, USA) using an YMC ODS3 column (50 mm × 4.6 mm, 5 ZM). Conditions were as follows: CH_3_CN/H_2_O eluent at 2.5 mL·min^−1^ flow [containing 0.1% trifluoroacetic acid (TFA)] at 35 °C, 8 min, gradient 5% CH_3_CN to 95% CH_3_CN, monitored by UV absorption at 214 nm and 254 nm. TLC analysis was carried out with glass precoated silica gel GF254 plates. TLC spots were visualized under UV light. Flash column chromatography was performed with a CombiFlash Rf system (Teledyne ISCO, Lincoln, NE, USA). All solvents and reagents were used directly as obtained commercially unless otherwise noted. Anhydrous dimethylformamide was purchased from Acros (Morris Plains, NJ, USA) and was used without further drying. All air and moisture sensitive reactions were carried out under an atmosphere of dry argon with heat-dried glassware and standard syringe techniques.

#### 3.1.1. Synthesis of 2-(2,6-Dichloro-3,5-dimethoxyphenyl)-4,4,5,5-tetramethyl-1,3,2-dioxaborolane (**6a**)

To a solution of 2-(3,5-dimethoxyphenyl)-4,4,5,5-tetramethyl-1,3,2-dioxaborolane (**6**, 15 g, 56.82 mmol) in DMF (300 mL), NCS (16.7 g, 125.0 mmol) was added and the mixture was stirred at 90 °C for 2 h. The reaction was cooled to 25 °C and quenched with distilled water. The solid product was filtered off, washed with water, and dried (92%). ^1^H-NMR (CDCl_3_) δ 6.56 (s, 1H), 3.91 (s, 6H), 1.45 (s, 12H); ^13^C-NMR (CDCl_3_) δ 153.73 (C × 2), 116.32, 98.39 (C × 2), 84.55 (C × 2), 56.13 (C × 2), 24.30 (C × 4); (+)ESI-MS *m*/*z* 334 [M + H]^+^.

#### 3.1.2. Synthesis of Methyl 6-(2,6-Dichloro-3,5-dimethoxyphenyl)-1*H*-indazole-4-carboxylate (**7**)

To a solution of methyl 6-bromo-1*H*-indazole-4-carboxylate (**5**, 10.0 g, 39.2 mmol) in 1,4-dioxane (300 mL), Boc_2_O (9.4 g, 43.1 mmol), Cs_2_CO_3_ (44.7 g, 137.2 mmol) were added into the reaction mixture, and the mixture was stirred at 25 °C for 0.5 h. 2-(2,6-dichloro-3,5-dimethoxyphenyl)-4,4,5,5-tetramethyl-1,3,2-dioxaborolane (**6a**) (14.4 g, 43.1 mmol), Pd(dppf)Cl_2_ (3.2 g, 3.92 mmol), H_2_O (100 mL) was added into the reaction mixture and stirred at 100 °C for 2 h under a nitrogen atmosphere. The reaction was cooled to 25 °C. The aqueous phase was extracted with dichloromethane, and the combined organic phase were washed with water and brine, dried over Na_2_SO_4_, filtered and concentrated in vacuo. The resultant residue was purified by column chromatography to get the intermediate as a white solid (62.3% yield). ^1^H-NMR (CDCl_3_) δ 8.67 (s, 1H), 7.86 (s, 1H), 7.62 (s, 1H), 6.69 (s, 1H), 4.04 (s, 3H), 4.01 (s, 6H); (+)ESI-MS *m*/*z* 382 [M + H]^+^.

#### 3.1.3. Synthesis of 6-(2,6-Dichloro-3,5-dimethoxyphenyl)-1*H*-indazole-4-carboxylic acid (**8**)

To a solution of 6-(2,6-dichloro-3,5-dimethoxyphenyl)-*N*-phenyl-1*H*-indazole-4-carboxamide (**7**, 8.9 g, 23.35 mmol) in THF (120 mL) lithium hydroxide (3.92 g, 93.44 mmol) dissolved in distilled water (30 mL) was added. Then, the mixture was stirred at 50 °C for 6 h. The reaction was cooled to 25 °C. The reaction mixture was acidified with 1N HCl and the solid product was filtered off, washed with water, and dried (yield: 76.2%). ^1^H-NMR (DMSO-*d*_6_) δ 8.45 (s, 1H), 7.67 (d, *J* = 3.8 Hz, 1H), 7.54 (s, 1H), 7.03 (s, 1H), 4.05–3.94 (m, 6H); ^13^C-NMR (CDCl_3_) δ 167.66, 154.10 (C × 2), 139.64, 134.83, 127.43, 127.34 (C × 2), 126.65, 126.27, 115.65, 114.23 (C × 2), 96.58, 56.15 (C × 2), (+)ESI-MS *m*/*z* 368 [M + H]^+^.

#### 3.1.4. Synthesis of 6-(2,6-Dichloro-3,5-dimethoxyphenyl)-*N*-phenyl-1*H*-indazole-4-carboxamides **10a**–**e**, **11a**–**e**, **12a**–**i**

To a solution of 6-(2,6-dichloro-3,5-dimethoxyphenyl)-1*H*-indazole-4-carboxylic acid (**8**, 30 mg, 0.082 mmol) in pyridine (3 mL) at 0 °C. POCl_3_ (12 μL, 0.090 mmol) was added, and the mixture was stirred at 0 °C for 1 h. Aniline was added into the reaction mixture and stirred at 0 °C for 10 min, then, the mixture was stirred at 25 °C for 1.5 h. The reaction was quenched with distilled water, and the organic phase was washed with water, 1N HCl and brine, dried over Na_2_SO_4_, filtered and concentrated in vacuo. The resultant residue was purified by column chromatography to get the final product as a white solid.

*6-(2,6-Dichloro-3,5-dimethoxyphenyl*)*-N-phenyl-1H-indazole-4-carboxamide* (**10a**). 82.3% yield; m.p. 115–117 °C; ^1^H-NMR (CDCl_3_) δ 8.70 (s, 1H), 8.18 (s, 1H), 7.72 (d, *J* = 8.0 Hz, 2H), 7.56 (s, 1H), 7.49 (s, 1H), 7.40 (t, *J* = 7.7, 7.7 Hz, 2H), 6.66 (s, 1H), 3.99 (s, 6H). ^13^C-NMR (CDCl_3_) δ 164.75, 154.64 (2 × C), 140.67, 139.94, 139.54, 137.76, 135.37, 135.21, 129.50, 129.14 (2 × C), 128.20, 124.73, 122.42, 121.71, 120.95, 120.35 (2 × C), 118.43, 118.38, 114.69, 114.56, 96.98, 56.64 (2 × CH_3_). C_22_H_17_Cl_2_N_3_O_3_ (+)ESI-MS *m*/*z* 442 [M + H]^+^.

*N-(3-Acetylphenyl*)*-6-(2,6-dichloro-3,5-dimethoxyphenyl*)*-1H-indazole-4-carboxamide* (**10b**). 78.4% yield; ^1^H-NMR (CDCl_3_) δ 8.69 (s, 1H), 8.30 (s, 1H), 8.19 (t, *J* = 2.0, 2.0 Hz, 1H), 8.08 (ddd, *J* = 8.1, 2.3, 1.0 Hz, 1H), 7.75 (ddd, *J* = 7.8, 1.7, 1.0 Hz, 1H), 7.58 (s, 1H), 7.52–7.47 (m, 2H), 6.65 (s, 1H), 3.98 (s, 6H), 2.63 (s, 3H). ^13^C-NMR (CDCl_3_) δ 198.05, 154.73 (2 × C), 140.67, 139.95, 137.85, 135.39, 134.91, 130.01, 129.72, 129.48, 124.96, 124.85, 124.43, 121.82, 120.81, 119.84, 119.73, 114.90, 114.62, 97.11, 56.69 (2 × CH_3_), 29.70. C_24_H_19_Cl_2_N_3_O_4_ (+)ESI-MS *m*/*z* 484 [M + H]^+^.

*N-(4-Acetylphenyl*)*-6-(2,6-dichloro-3,5-dimethoxyphenyl*)*-1H-indazole-4-carboxamide* (**10c**). 76.3% yield; ^1^H-NMR (DMSO-*d*_6_) δ 13.50 (s, 1H), 10.62 (s, 1H), 8.51 (s, 1H), 7.99 (s, 4H), 7.73 (s, 1H), 7.66 (s, 1H), 7.06 (s, 1H), 4.00 (s, 6H), 2.56 (s, 3H). ^13^C-NMR (DMSO-*d*_6_) δ 197.12, 165.45, 154.96 (2 × C), 143.88, 140.02, 134.53, 134.33, 132.55, 130.10, 129.72 (2 × C), 127.20, 122.52, 122.90, 120.09 (2 × C), 116.85, 115.31, 113.61, 98.59, 57.29 (2 × CH_3_), 26.96. C_24_H_19_Cl_2_N_3_O_4_ (+)ESI-MS *m*/*z* 484 [M + H]^+^.

*6-(2,6-Dichloro-3,5-dimethoxyphenyl*)*-N-(3-methoxyphenyl*)*-1H-indazole-4-carboxamide* (**10d**). 78.6% yield; ^1^H-NMR (CDCl_3_) δ 8.68 (s, 1H), 8.10 (s, 1H), 7.55 (s, 1H), 7.46 (d, *J* = 2.1 Hz, 2H), 7.18 (d, *J* = 8.3 Hz, 1H), 6.72 (ddd, *J* = 8.2, 2.6, 1.0 Hz, 1H), 6.64 (s, 1H), 3.97 (s, 6H), 3.83 (s, 3H). ^13^C-NMR (CDCl_3_) δ 164.68, 160.27, 154.69 (2 × C), 140.66, 139.90, 138.96, 135.48, 135.31, 129.82 (2 × C), 128.27, 121.76, 120.93, 114.60 (2 × C), 112.38, 110.78, 105.84, 97.04, 56.66 (2 × CH_3_), 55.41. C_23_H_19_Cl_2_N_3_O_4_ (+)ESI-MS *m*/*z* 472 [M + H]^+^.

*6-(2,6-Dichloro-3,5-dimethoxyphenyl*)*-N-(4-methoxyphenyl*)*-1H-indazole-4-carboxamide* (**10e**). 72.5% yield; ^1^H-NMR (CDCl_3_) δ 8.69 (s, 1H), 7.89 (s, 1H), 7.60 (d, *J* = 8.9 Hz, 3H), 7.58 (d, *J* = 1.2 Hz, 1H), 7.47–7.45 (m, 1H), 6.95 (d, *J* = 8.9 Hz, 2H), 6.69 (s, 1H), 4.01 (s, 6H), 3.85 (d, *J* = 1.0 Hz, 3H). ^13^C-NMR (CDCl_3_) δ 164.61, 156.77, 154.72 (2 × C), 140.65, 139.98, 135.44, 130.76, 130.02, 129.74, 128.36, 122.21 (2 × C), 121.64, 120.98, 114.65, 114.33 (2 × C), 97.07, 56.69 (2 × CH_3_), 55.54. C_22_H_18_Cl_2_N_4_O_4_ (+)ESI-MS *m*/*z* 473 [M + H]^+^.

*6-(2,6-Dichloro-3,5-dimethoxyphenyl*)*-N-(6-methoxypyridin-2-yl*)*-1H-indazole-4-carboxamide* (**11a**). 71.5% yield; ^1^H-NMR (CDCl_3_) δ 8.72 (d, *J* = 1.2 Hz, 1H), 8.46 (s, 1H), 8.00 (d, *J* = 7.8 Hz, 1H), 7.70 (t, *J* = 8.0, 8.0 Hz, 1H), 7.62 (s, 1H), 7.54 (d, *J* = 1.2 Hz, 1H), 6.70 (s, 1H), 6.57 (d, *J* = 8.1 Hz, 1H), 4.02 (s, 6H), 3.91 (d, *J* = 1.2 Hz, 3H). ^13^C-NMR (CDCl_3_) δ 164.96, 162.93, 154.65 (2 × C), 149.00, 140.99, 140.77, 140.01, 135.31, 134.21, 127.41, 121.97 (2 × C), 120.45, 115.38, 114.62, 106.16, 106.00, 97.10, 56.63 (2 × CH_3_), 53.50. C_22_H_18_Cl_2_N_4_O_4_ (+^)^ESI-MS *m*/*z* 473 [M + H]^+^.

*6-(2,6-Dichloro-3,5-dimethoxyphenyl*)*-N-(2-methoxypyridin-4-yl*)*-1H-indazole-4-carboxamide* (**11b**). 72.2% yield; ^1^H-NMR (CDCl_3_) δ 8.68 (s, 1H), 8.15 (d, *J* = 5.7 Hz, 1H), 8.05 (s, 1H), 7.62 (s, 1H), 7.46 (s, 1H), 7.22 (s, 1H), 7.20 (d, *J* = 5.9 Hz, 1H), 6.70 (s, 1H), 4.02 (s, 6H), 3.98 (s, 3H). ^13^C-NMR (CDCl_3_) δ 165.54, 164.88, 154.68 (2 × C), 147.75 (2 × C), 146.94, 140.68, 139.69, 135.41, 135.22, 127.35, 121.91, 120.93, 115.23, 114.49, 108.39, 99.85, 97.01, 56.64 (2 × CH_3_), 53.68. C_22_H_18_Cl_2_N_4_O_4_ (+)ESI-MS *m*/*z* 473 [M + H]^+^.

*6-(2,6-Dichloro-3,5-dimethoxyphenyl*)*-N-(4-methoxypyridin-2-yl*)*-1H-indazole-4-carboxamide* (**11c**). 68.2% yield; ^1^H-NMR (CDCl_3_) δ 8.72 (s, 1H), 8.12 (d, *J* = 6.4 Hz, 1H), 8.10 (d, *J* = 1.6 Hz, 1H), 7.61 (s, 1H), 7.54 (s, 1H), 6.70 (s, 1H), 6.67 (dd, *J* = 6.0, 1.8 Hz, 1H), 4.02 (s, 6H), 3.97 (s, 3H). ^13^C-NMR (CDCl_3_) δ 167.67, 164.98, 154.70 (2 × C), 153.10, 148.41, 140.68, 139.83, 135.62, 135.26, 127.54, 123.47, 122.34, 120.90, 114.96, 114.69, 108.05, 98.94, 97.25, 56.71, 55.52 (2 × CH_3_). C_22_H_18_Cl_2_N_4_O_4_ (+)ESI-MS *m*/*z* 473 [M + H]^+^.

*6-(2,6-Dichloro-3,5-dimethoxyphenyl*)*-N-(6-methoxypyrimidin-4-yl*)*-1H-indazole-4-carboxamide* (**11d**). 65.5% yield; ^1^H-NMR (CDCl_3_) δ 8.71 (s, 21H), 8.52 (s, 1H), 7.83 (s, 2H), 7.64 (s, 1H), 7.52 (s, 1H), 6.71 (s, 1H), 4.05 (s, 3H), 4.02 (s, 6H). ^13^C-NMR (CDCl_3_) δ 171.44, 165.06, 157.70, 157.58, 154.77 (2 × C), 140.71, 139.60, 135.59, 135.30, 126.67, 122.39, 120.87, 115.43, 114.63, 97.28, 95.35, 56.72 (2 × C), 54.30, 53.43. C_21_H_17_Cl_2_N_5_O_4_ (+)ESI-MS *m*/*z* 474 [M + H]^+^.

*6-(2,6-Dichloro-3,5-dimethoxyphenyl*)*-N-(2-methoxypyrimidin-4-yl*)*-1H-indazole-4-carboxamide* (**11e**). 66.6% yield; ^1^H-NMR (CDCl_3_) δ 8.75 (s, 1H), 8.41 (d, *J* = 5.7 Hz, 1H), 7.63 (s, 1H), 7.52 (s, 1H), 6.70 (s, 1H), 6.52 (d, *J* = 5.8 Hz, 1H), 4.01 (s, 6H), 3.99 (s, 3H). ^13^C-NMR (CDCl_3_) δ 170.41, 163.90, 158.20, 157.11, 154.74 (2 × C), 140.70, 139.84, 135.57, 135.42, 127.55, 122.29, 121.15, 115.19, 114.68 (2 × C), 104.12, 97.15, 56.70 (2 × CH_3_), 54.04. C_21_H_17_Cl_2_N_5_O_4_ (+)ESI-MS *m*/*z* 474 [M + H]^+^.

*Methyl 3-(6-(2,6-dichloro-3,5-dimethoxyphenyl*)*-1H-indazole-4-carboxamido*)*benzoate* (**12a**). 63.5% yield; ^1^H-NMR (CDCl_3_) δ 8.70 (s, 1H), 8.20 (s, 1H), 8.11 (d, *J* = 8.3 Hz, 1H), 8.04 (s, 1H), 7.88 (d, *J* = 8.0 Hz, 1H), 7.62 (s, 1H), 7.53–7.47 (m, 2H), 6.71 (s, 1H), 4.02 (s, 6H), 3.96 (s, 3H). ^13^C-NMR (CDCl_3_) δ 166.67, 164.81, 154.71 (2 × C), 140.70, 139.84, 138.01, 135.46, 135.28, 131.04, 130.02, 129.73, 129.34, 127.82, 125.75, 124.76, 121.84, 121.12, 114.84, 114.58, 97.08, 56.67 (2 × CH_3_), 52.30. C_24_H_19_Cl_2_N_3_O_5_ (+)ESI-MS *m*/*z* 500 [M + H]^+^.

*6-(2,6-Dichloro-3,5-dimethoxyphenyl*)*-N-(3-(methylcarbamoyl*)*phenyl*)*-1H-indazole-4-carboxamide* (**12b**). 62.4% yield; m.p. 155–157 °C; ^1^H-NMR (CDCl_3_) δ 8.68 (s, 1H), 8.17 (s, 2H), 7.85 (d, *J* = 8.3 Hz, 1H), 7.61 (d, *J* = 8.9 Hz, 2H), 7.49 (d, *J* = 4.6 Hz, 1H), 6.70 (s, 1H), 4.02 (s, 6H), 3.05 (d, *J* = 4.8 Hz, 3H). ^13^C-NMR (CDCl_3_) δ 167.78, 164.97, 154.70 (2 × C), 140.71, 139.83, 138.19, 135.52, 135.46, 135.11, 129.49, 127.71, 123.08, 122.96, 122.06, 120.81, 118.56 (2 × C), 114.89, 114.58, 97.09, 56.67 (2 × CH_3_), 26.92. C_24_H_20_Cl_2_N_4_O_4_ (+)ESI-MS *m*/*z* 499 [M + H]^+^.

*Ethyl 3-(6-(2,6-dichloro-3,5-dimethoxyphenyl*)*-1H-indazole-4-carboxamido*)*benzoate* (**12c**). 61.5% yield; ^1^H-NMR (CDCl_3_) δ 8.71 (s, 1H), 8.29 (s, 1H), 8.19 (t, *J* = 1.9, 1.9 Hz, 1H), 8.15 (d, *J* = 8.4 Hz, 1H), 7.86 (dt, *J* = 7.8, 1.4, 1.4 Hz, 1H), 7.59 (d, *J* = 1.2 Hz, 1H), 7.52–7.46 (m, 2H), 6.66 (s, 1H), 4.40 (q, *J* = 7.3, 7.3, 7.3 Hz, 2H), 3.99 (s, 6H), 1.41 (t, *J* = 7.1, 7.1 Hz, 3H). ^13^C-NMR (CDCl_3_) δ 166.13, 164.78, 154.74 (2 × C), 139.84, 137.91, 135.51, 131.42, 129.32 (2 × C), 127.87, 125.76, 125.02, 124.71 (2 × C), 121.86, 121.06, 114.76, 114.62 (2 × C), 97.12, 61.23, 56.69 (2 × CH_3_), 14.34. C_25_H_21_Cl_2_N_3_O_5_ (+)ESI-MS *m*/*z* 514 [M + H]^+^.

*6-(2,6-Dichloro-3,5-dimethoxyphenyl*)*-N-(3-nitrophenyl*)*-1H-indazole-4-*carboxamide (**12d**). 62.2% yield; ^1^H-NMR (DMSO-*d*_6_) δ 13.51 (s, 1H), 10.75 (s, 1H), 8.83 (s, 1H), 8.54 (s, 1H), 8.27 (d, *J* = 8.2 Hz, 1H), 7.99 (d, *J* = 8.6 Hz, 1H), 7.75 (s, 1H), 7.70–7.65 (m, 2H), 7.07 (s, 1H), 4.00 (s, 6H). ^13^C-NMR (DMSO-*d*_6_) δ 165.56, 154.97 (2 × C), 148.35, 140.86, 140.67, 140.00, 134.59, 134.44, 130.50, 126.87, 126.79, 122.48, 120.92, 118.67, 115.43, 115.02, 113.62, 98.61, 57.30, 55.37 (C × CH_3_). C_22_H_16_Cl_2_N_4_O_5_ (+)ESI-MS *m*/*z* 487 [M + H]^+^.

*6-(2,6-Dichloro-3,5-dimethoxyphenyl*)*-N-(3-(3-methyl-2-oxoimidazolidin-1-yl*)*phenyl*)*-1H-indazole-4-carboxamide* (**12e**). 63.3% yield; ^1^H-NMR (CDCl_3_) δ 8.48 (d, *J* = 1.0 Hz, 1H), 8.28 (s, 1H), 8.00 (t, *J* = 2.1, 2.1 Hz, 1H), 7.57–7.53 (m, 2H), 7.49 (s, 1H), 7.45 (d, *J* = 1.1 Hz, 1H), 7.32 (t, *J* = 8.2, 8.2 Hz, 1H), 7.22 (dd, *J* = 7.9, 1.6 Hz, 2H), 6.65 (s, 1H), 3.98 (s, 6H), 3.89–3.83 (m, 2H), 3.52–3.47 (m, 2H), 2.90 (s, 3H). ^13^C-NMR (CDCl_3_) δ 164.92, 158.18, 154.66 (2 × C), 141.11, 140.66, 140.00, 138.50, 135.46, 135.05, 129.46, 128.28, 121.99, 120.75, 114.71, 114.52, 114.31, 113.12, 109.33 (2 × C), 97.15, 56.69 (2 × CH_3_), 42.43, 31.22, 29.70. C_26_H_23_Cl_2_N_5_O_4_ (+)ESI-MS *m*/*z* 540 [M + H]^+^.

*6-(2,6-Dichloro-3,5-dimethoxyphenyl*)*-N-(3-morpholinophenyl*)*-1H-*indazole*-4-carboxamide* (**12f**). 64.8% yield; m.p. 159–161 °C; ^1^H-NMR (CDCl_3_) δ 8.69 (s, 1H), 8.07 (s, 1H), 7.57 (s, 1H), 7.52 (t, *J* = 2.1, 2.1 Hz, 1H), 7.47 (d, *J* = 1.1 Hz, 1H), 7.06 (dd, *J* = 8.2, 1.2 Hz, 1H), 6.73 (dd, *J* = 8.2, 2.4 Hz, 1H), 6.67 (s, 1H), 4.00 (s, 6H), 3.91–3.85 (m, 4H), 3.25–3.19 (m, 4H). ^13^C-NMR (CDCl_3_) δ 164.75, 154.64 (2 × C), 152.08, 140.67, 139.93, 138.79, 135.39, 135.23, 129.66 (2 × C), 128.33, 121.70, 120.91, 114.63, 114.54, 111.94, 111.64, 107.60, 96.97, 66.88 (2 × CH_2_), 56.63 (2 × CH_3_), 49.17 (2 × CH_2_). C_26_H_24_Cl_2_N_4_O_4_ (+)ESI-MS *m*/*z* 527 [M + H]^+^.

*6-(2,6-Dichloro-3,5-dimethoxyphenyl*)*-N-(3-(piperazin-1-yl*)*phenyl*)*-1H-indazole-4-*carboxamide(**12g**). 60.5% yield; m.p. 221–223 °C; ^1^H-NMR (DMSO-*d*_6_) δ 13.48 (s, 1H), 10.21 (s, 1H), 8.49 (s, 1H), 7.67 (s, 1H), 7.63 (s, 1H), 7.51 (s, 1H), 7.36 (d, *J* = 8.0 Hz, 1H), 7.24 (t, *J* = 8.0, 8.0 Hz, 1H), 7.06 (s, 1H), 6.78 (d, *J* = 8.2 Hz, 1H), 4.00 (s, 6H), 3.23 (s, 4H), 3.17 (d, *J* = 5.2 Hz, 4H). ^13^C-NMR (CDCl_3_) δ 154.60 (2 × C), 150.89, 140.75, 139.27, 139.06, 135.25, 130.09, 129.98, 129.76 (2 × C), 128.04, 121.75, 120.56, 115.03, 114.51, 114.00, 113.17, 109.09, 97.00, 56.6 (2 × CH_3_), 47.36 (2 × CH_2_), 43.77 (2 × CH_2_). C_26_H_25_Cl_2_N_5_O_3_ (+)ESI-MS *m*/*z* 526 [M + H]^+^.

*6-(2,6-Dichloro-3,5-dimethoxyphenyl*)*-N-(3-(1-methyl-1H-pyrazol-4-yl*)*phenyl*)*-1H-indazole-4-carboxamide* (**12h**). 68.8% yield; m.p. 188–190 °C; ^1^H-NMR (CDCl_3_) δ 8.29 (s, 1H), 7.91 (s, 1H), 7.78 (s, 1H), 7.67 (s, 1H), 7.56 (s, 1H), 7.52 (s, 2H), 7.39 (m, 1H), 7.36 (d, *J* = 7.9 Hz, 1H), 3.97 (s, 6H), 3.94 (s, 3H). ^13^C-NMR (DMSO-*d*_6_) δ 165.10, 154.95 (2 × C), 140.86, 140.14, 139.90, 136.35, 134.59, 133.42, 131.09, 129.54, 129.00, 128.90, 128.28, 127.66, 122.33, 122.23, 121.06, 118.79, 117.59, 114.88, 113.63, 98.55, 57.29 (2 × CH_3_), 39.12. C_26_H_21_Cl_2_N_5_O_3_ (+)ESI-MS *m*/*z* 522 [M + H]^+^.

*6-(2,6-Dichloro-3,5-dimethoxyphenyl*)*-N-(3-(pyrimidin-5-yl*)*phenyl*)*-1H-indazole-4-carboxamide* (**12i**). 67.6% yield; m.p. 177–179 °C; ^1^H-NMR (CDCl_3_) δ 9.20 (s, 1H), 8.97 (s, 2H), 8.69 (s, 1H), 8.40 (s, 1H), 8.05 (s, 1H), 7.79–7.73 (m, 1H), 7.57 (s, 1H), 7.53 (d, *J* = 7.9 Hz, 1H), 7.50 (s, 1H), 7.36 (d, *J* = 7.7 Hz, 1H), 3.96 (s, 6H). ^13^C-NMR (DMSO-*d*_6_) δ 165.25, 157.86, 155.15 (2 × C), 154.96 (2 × C), 140.86, 140.34, 140.10, 134.66, 134.59, 134.50, 133.79, 130.16, 127.44, 122.82, 122.29, 121.31, 120.96, 119.43, 115.05, 113.63 (2 × C), 98.56,57.29 (2 × CH_3_). C_26_H_19_Cl_2_N_5_O_3_ (+)ESI-MS *m*/*z* 520 [M + H]^+^.

#### 3.1.5. Synthesis of 6-(2,6-Dichloro-3,5-dimethoxyphenyl)-*N*-(3-(4-piperazin-1-yl)phenyl-1*H*-indazole-4-carboxamides **13a**–**e**

To a solution of 6-(2,6-dichloro-3,5-dimethoxyphenyl)-1*H*-indazole-4-carboxylic acid (**8**) in dry DCM, DIPEA and HATU were added and the mixture was stirred at 25 °C for 4 h. The appropriate 3-(4-ethylpiperazin-1-yl)aniline **9a**–**e** was then added and the mixture was stirred at 25 °C for 0.5 h. The reaction was quenched with distilled water, and the organic phase was washed with water and brine, dried over Na_2_SO_4_, filtered and concentrated in vacuo.

*6-(2,6-Dichloro-3,5-dimethoxyphenyl*)*-N-(3-(4-methylpiperazin-1-yl*)*phenyl*)*-1H-*indazole*-4-carboxamide* (**13a**). 60.7% yield; m.p. 205–207 °C; ^1^H-NMR (CDCl_3_) δ 8.68 (s, 1H), 7.92 (s, 1H), 7.59 (s, 1H), 7.48 (s, 1H), 7.46 (s, 1H), 7.05 (d, *J* = 8.8 Hz, 1H), 6.76 (d, *J* = 8.5 Hz, 1H), 6.69 (s, 1H), 4.02 (s, 6H), 3.39–3.31 (m, 4H), 2.77–2.68 (m, 4H), 2.46 (s, 3H). C_27_H_27_Cl_2_N_5_O_3_ (+)ESI-MS *m*/*z* 540 [M + H]^+^.

*6-(2,6-Dichloro-3,5-dimethoxyphenyl*)*-N-(3-(4-ethylpiperazin-1-yl*)*phenyl*)*-1H-indazole-4-carboxamide* (**13b**). 60.3% yield; ^1^H-NMR (CDCl_3_) δ 8.67 (s, 1H), 7.97 (s, 1H), 7.60 (s, 1H), 7.49 (s, 1H), 7.46 (s, 1H), 7.07 (dd, *J* = 8.5, 1.4 Hz, 1H), 6.74 (dd, *J* = 8.6, 2.3 Hz, 1H), 6.69 (s, 1H), 4.01 (s, 6H), 3.47–3.38 (m, 4H), 2.83 (s, 4H), 2.74–2.65 (m, 2H), 0.90 (t, *J* = 6.8, 6.8 Hz, 3H). ^13^C-NMR (CDCl_3_) δ 164.72, 154.72 (2 × C), 140.65, 139.93, 138.73, 135.48, 135.26, 130.49, 129.74 (2 × C), 128.36, 121.76, 120.90, 114.64, 114.53 (2 × C), 112.43, 111.77, 108.23, 97.08, 67.10, 56.69 (2 × C), 52.33, 52.24, 48.18, 14.13. C_28_H_29_Cl_2_N_5_O_3_ (+)ESI-MS *m*/*z* 554 [M + H]^+^.

*N-(3-(4-Allylpiperazin-1-yl*)*phenyl*)*-6-(2,6-dichloro-3,5-dimethoxyphenyl*)*-1H-indazole-4-carboxamide* (**13c**). 56.2% yield; ^1^H-NMR (CDCl_3_) δ 8.68 (s, 1H), 7.92 (s, 1H), 7.59 (s, 1H), 7.46 (s, 2H), 7.10–7.02 (m, 1H), 6.75 (d, *J* = 8.5 Hz, 1H), 6.72 (s, 1H), 5.96 (m, 1H),5.30–5.22 (m, 2H), 4.05–3.99 (m, 6H), 3.39–3.31 (m, 4H), 3.21–3.12 (m, 2H), 2.78–2.67 (m, 4H). C_29_H_29_Cl_2_N_5_O_3_ (+)ESI-MS *m*/*z* 566 [M + H]^+^.

*6-(2,6-Dichloro-3,5-dimethoxyphenyl*)*-N-(3-(4-isopropylpiperazin-1-yl*)phenyl)*-1H-indazole-4*-*carboxamide* (**13d**). 55.6% yield; ^1^H-NMR (CDCl_3_) δ 8.67 (s, 1H), 7.97 (s, 1H), 7.60 (s, 1H), 7.49 (s, 1H), 7.01 (t, *J* = 8.0, 8.0 Hz, 1H), 6.43–6.35 (m, 2H), 6.32 (d, *J* = 8.0 Hz, 1H), 3.99 (s, 6H), 3.47–3.38 (m, 4H), 2.48 (s, 4H), 1.31 (s, 1H), 1.26 (d, *J* = 6.6 Hz, 6H). C_29_H_31_Cl_2_N_5_O_3_ (+)ESI-MS *m*/*z* 568 [M + H]^+^.

*N-(3-(4-Acetylpiperazin-1-yl*)*phenyl*)*-6-(2,6-dichloro-3,5-dimethoxyphenyl*)*-1H-indazole-4-carboxamide* (**13e**). 65.8% yield; m.p. 209–211 °C; ^1^H-NMR (CDCl_3_) δ 8.68 (s, 1H), 8.06 (s, 1H), 7.59 (s, 1H), 7.54 (s, 1H), 7.47 (s, 1H), 7.06 (d, *J* = 7.6 Hz, 1H), 6.75 (d, *J* = 10.9 Hz, 1H), 6.68 (s, 1H), 4.01 (s, 6H), 3.81–3.76 (m, 2H), 3.67–3.62 (m, 2H), 3.51 (s, 3H),3.30–3.24 (m, 2H), 3.24–3.19 (m, 1H). ^13^C-NMR (CDCl_3_) δ 169.10, 164.75, 154.71 (2 × C), 151.71, 140.67, 139.92, 138.81, 135.46, 135.27, 129.75, 128.30, 121.75, 120.91, 114.59 (2 × C), 112.75, 111.99, 108.48, 97.05, 56.68 (2 × CH_3_), 49.43, 49.18, 46.18, 41.33, 21.37. C_28_H_27_Cl_2_N_5_O_4_ (+)ESI-MS *m*/*z* 568 [M + H]^+^.

### 3.2. Molecular Docking

The structure of human FGFR1 kinase domain with its inhibitor NVP-BGJ398 (ID: 3TT0) was downloaded from PDB database. Two compounds (**4** and **10a**) were docked into FGFR1 ATP site using Glide with XP mode. The protein structure was prepared using Protein preparation wizard tool in Maestro, which fixed the protein structure by verifying proper assignment of bonds, adding hydrogens, deleting all water molecules and refining hydrogen atoms. The binding pattern of NVP-BGJ398 to FGFR1 was used as reference to define the active site and docking grids within a 14 × 14 × 14 Å box. All the docking parameters were set as default values in the Glide software (Schrodinger Company, Cambridge, MA, USA). Only the best scored conformation was selected for visualization with Pymol software (Schrodinger Company). 

### 3.3. Elisa Kinase Assay

The effects of compounds on the activities of FGFR1 kinases were determined using enzyme-linked immunosorbent assays (ELISAs) with purified recombinant proteins. Briefly, 20 μg/mL poly (Glu, Tyr)_4:1_ (Sigma, St. Louis, MO, USA) was pre-coated in 96-well plates as a substrate. A 50-μL aliquot of 10 μmol/L ATP solution diluted in kinase reaction buffer (50 mmol/L HEPES [pH 7.4], 50 mmol/L MgCl_2_, 0.5 mmol/L MnCl_2_, 0.2 mmol/L Na_3_VO_4_, and 1 mmol/L DTT) was added to each well; 1 μL of various concentrations of compounds diluted in 1% DMSO (*v*/*v*) (Sigma) were then added to each reaction well. DMSO (1%, *v*/*v*) was used as the negative control. The kinase reaction was initiated by the addition of purified tyrosine kinase proteins diluted in 49 μL of kinase reaction buffer. After incubation for 60 min at 37 °C, the plate was washed three times with phosphate-buffered saline (PBS) containing 0.1% Tween 20 (T-PBS). Anti-phosphotyrosine (PY99) antibody (100 μL; 1:500, diluted in 5 mg/mL BSA T-PBS) was then added. After a 30-min incubation at 37 °C, the plate was washed three times, and 100 μL horseradish peroxidase-conjugated goat anti-mouse IgG (1:2000, diluted in 5 mg/mL BSA T-PBS) was added. The plate was then incubated at 37 °C for 30 min and washed 3 times. A 100-μL aliquot of a solution containing 0.03% H_2_O_2_ and 2 mg/mL *o*-phenylenediamine in 0.1 mol/L citrate buffer (pH 5.5) was added. The reaction was terminated by the addition of 50 μL of 2 mol/L H_2_SO_4_ as the color changed, and the plate was analyzed using a multi-well spectrophotometer (SpectraMAX 190, Molecular Devices, Palo Alto, CA, USA) at 490 nm. The inhibition rate (%) was calculated using the following equation: [1 − (*A*_490_/*A*_490 control_)] × 100%. The IC_50_ values were calculated from the inhibition curves in two separate experiments.

## 4. Conclusions

In summary, we have reported the design, synthesis, and biological evaluation of a novel series of 6-(2,6-dichloro-3,5-dimethoxyphenyl)-4-Substituted-1*H*-indazole derivatives as potent FGFR kinase inhibitors. Through four rounds of optimization, compound **13a** stood out as the most potent FGFR1 inhibitor with an inhibition potency IC_50_ = 30.2 ± 1.9 nM). We think current study not only suggests new potent FGFR inhibitors for the scientific community, but also points out a new direction for optimizing this promising scaffold.

## Figures and Tables

**Table 1:** Structures and activities of indazole derivatives **10a**–**e**. 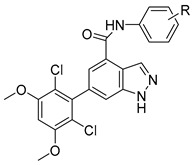

Compound No.	R	Enzyme Inhibition (%) ^a^	
		1 μmol/L	0.1 μmol/L
**10a**	H	84.1	58.8
**10b**	*m*-acetyl	82.4	76.4
**10c**	*p*-acetyl	52.9	32.2
**10d**	*m*-methoxyl	78.4	57.0
**10e**	*p*-methoxyl	68.5	11.9
**AZD4547**	/	94.3	93.2

^a^ The IC_50_ value of AZD4547 is 1.8 ± 0.1 nM (mean ± SD), which was significantly higher than that of any of the new compounds tested.

**Table 2:** Structures and activities of indazole derivatives **11a**–**e**. 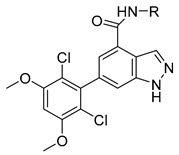

Compound No.	R	Enzyme Inhibition (%)	
		1 μmol/L	0.1 μmol/L
**10d**	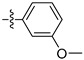	78.4	57.0
**11a**	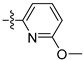	79.2	47.2
**11b**	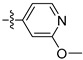	84.3	49.5
**11c**	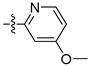	80	42.3
**11d**	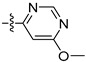	78.5	43.1
**11e**	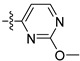	57.4	31

**Table 3:** Structures and activities of indazole derivatives **12a**–**i**. 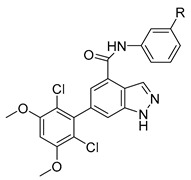

Compound No.	R	Enzyme Inhibition (%)		IC_50_ (nM) ^a^
		0.1 μmol/L	0.01 μmol/L	
**10a**	H	58.8	27.3	69.1 ± 19.8
**12a**	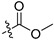	35.7	17.1	/
**12b**	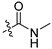	62.3	42.1	38.6 ± 0.2
**12c**	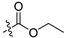	36.8	9.9	/
**12d**	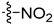	31.6	17.4	/
**12e**	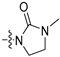	36.7	17.2	/
**12f**	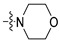	58.7	28.3	54.0 ± 8.7
**12g**	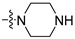	60.9	45.1	78.8 ± 14.2
**12h**	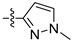	52.7	34.5	102.9 ± 0.6
**12i**	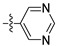	60.3	35.1	86.2 ± 17.0

^a^ The IC_50_ or inhibition values shown as the mean ± SD (nM) are calcuated from two separate experiments.

**Table 4:** Structures and activities of indazole derivatives **13a**–**e**. 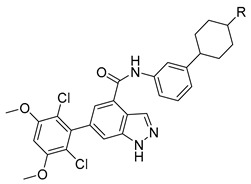

Compound No.	R	Enzyme Inhibition (%)		IC_50_ (nM) ^a^
		0.1 μmol/L	0.01 μmol/L	
**12g**	H	60.9	45.1	78.8 ± 14.2
**13a**	–CH_3_	72.7	48.3	30.2 ± 1.9
**13b**	–C_2_H_5_	26.4	13.7	328.4 ± 65.7
**13c**	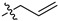	41.3	35.7	463.9 ± 99.1
**13d**	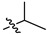	18.5	7.2	/
**13e**	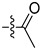	64.7	27.7	117.9 ± 3.9

^a^ The IC_50_ or inhibition values shown as the mean ± SD (nM) are calcuated from two separate experiments.
